# Mother and father depression symptoms and child emotional difficulties: a network model

**DOI:** 10.1192/bjp.2023.8

**Published:** 2023-05

**Authors:** Alex F. Martin, Barbara Maughan, Deniz Konac, Edward D. Barker

**Affiliations:** Department of Psychology, Institute of Psychiatry, Psychology & Neuroscience, King's College London, UK; Social, Genetic & Developmental Psychiatry Centre, Institute of Psychiatry, Psychology and Neuroscience, King's College London, UK; Department of Psychology, Institute of Psychiatry, Psychology & Neuroscience, King's College London, UK and Department of Psychology, Adana Alparslan Turkes Science and Technology University, Turkey

**Keywords:** ALSPAC, co-occurrence, within-family transmission, psychopathology, treatment targets

## Abstract

**Background:**

Mother and father depression symptoms often co-occur, and together can have a substantial impact on child emotional well-being. Little is understood about symptom-level mechanisms underlying the co-occurrence of depression symptoms within families.

**Aims:**

The objective was to use network analysis to examine depression symptoms in mothers and fathers after having a baby, and emotional symptoms in children in early adolescence.

**Method:**

We examined data from 4492 mother–father–child trios taken from a prospective, population-based cohort in the UK. Symptoms were examined using two unregularised partial correlation network models. The initial model was used to examine the pattern of associations, i.e. the overall network structure, for mother and father depression symptoms, and then to identify bridge symptoms that reinforce depression symptoms between parents during offspring infancy. The second model examined associations between the parent symptom network, including bridge symptoms, with later child emotional difficulties.

**Results:**

The study included 4492 mother–father–child trios; 2204 (49.1%) children were female. Bridge symptoms reinforcing mother and father depression symptoms were feeling guilty and self-harm ideation. For mothers, the bridge symptom of feeling guilty, and symptoms of anhedonia, panic and sadness were highly connected with child emotional difficulties. For fathers, the symptom of feeling overwhelmed associated with child emotional difficulties. Guilt and anhedonia in fathers appeared to indirectly associate with child emotional difficulties through the same symptom in mothers.

**Conclusions:**

Our findings suggest that specific symptom cascades are central for co-occurring depression in parents and increased vulnerability in children, providing potential therapeutic targets.

Depression is common in parents of infants, with one in ten mothers and fathers experiencing clinical levels of symptomatology,^1,2^ and one in two experiencing subclinical symptoms.^3^ Depression in parents often co-occurs, with up to 50% of mothers and fathers experiencing symptoms at the same time.^4^ Parental depression is reported to be one of the strongest risk factors for emotional difficulties in children,^5^ and when depression symptoms co-occur, this can further increase risk compared with depression in one parent.^6^ The association between parental depression during infancy and emotional difficulties in children is long lasting,^7^ and effects persist over and above changes in risk factors with the transition to adolescence a vulnerable period for the emergence of symptoms in children.^8^

However, little is understood about symptom-level mechanisms that may help to explain co-occurrence of depression symptoms between parents and the association with emotional difficulties in children.

## Using network analysis to investigate depression within families

Most studies investigating the co-occurrence of depression in parents use summed symptoms or ‘clinical cut-off’ scores.^9^ This approach assumes that all symptoms are equally important, but this may not be the case. Network analysis provides a framework to investigate symptom-level associations, where symptom patterns or clusters of cognitions and behaviours, can influence each other.^10^ These symptom clusters can be conceptualised as feedback loops driving depressive processes, for example, insomnia can cause fatigue, which can cause psychomotor-related symptoms, which in turn can disrupt sleep.^10,11^

The network approach can also inform the understanding of symptom-level mechanisms underlying the co-occurrence of depression in parents.^2,4^ There is good reason to pursue this research aim: one study found associations between mothers and fathers for only some depression symptoms, specifically insomnia, feeling guilty and self-harm ideation.^12^ These symptoms may act as ‘bridges’, providing connections and activating symptoms, between parents.^13,14^

Network models can also be used to examine depression-related risk pathways between parents and children.^15^ Findings from an intervention study in this area suggest that the parental symptoms of anhedonia (the inability to feel pleasure) and impaired attention may be important in the intergenerational transmission of psychopathology,^16^ in part because they may be associated with more withdrawn and less nurturing parenting, both of which are associated with emotional symptoms in children.^17^

## Study aims and hypotheses

Examining relationships between depression symptoms in mothers and fathers during infancy and emotional difficulties in children may provide important insights beyond existing studies of overall symptom severity. Network models provide a framework for examining relationships between symptoms and can provide useful clinical insights, whereby activating symptoms between family members could be targeted for more rapid recovery.^18^ Against this background, the aims of the current study are first, to examine the overall network structure of mother and father depression symptoms during infancy; second, to identify bridge symptoms that provide a pathway between mother and father symptoms, reinforcing and activating the symptom networks; and third, to examine whether the bridge symptoms and other symptoms within the network associate with emotional difficulties in the child, at the transition to adolescence.

This is the first study to examine the network structure of depression symptoms within families. Despite the novelty of our methodology, we were able to draw on existing research to make the following predictions.

- We expected that symptoms previously found to constitute an underlying factor of anxiety-related depression symptoms would be highly interrelated (i.e. cluster) in our network.^19^
- Based on previous findings,^12^ we hypothesised that insomnia, feeling guilty and self-harm ideation would represent bridges between mother and father symptoms.
- The association between parent depression and child internalising psychopathology is well-established as larger between mothers and children compared with fathers and children.^7^ Therefore, we expected that more mother than father symptoms would be associate with child emotional difficulties.

As a result of the paucity of research testing associations between individual depression symptoms in families, we did not make predictions about the effects of specific symptoms between parents and children.

## Method

### Participants

Our study comprised participants from an ongoing epidemiological study, the Avon Longitudinal Study of Parents and Children (ALSPAC).^20,21^ Pregnant women resident in Avon, UK with expected dates of delivery 1 April 1991 to 31 December 1992 were invited to take part. The initial number of women enrolled was 14 541, resulting in 13 988 children alive at 1 year of age. The ALSPAC cohort is broadly representative of the general population in the UK. The study website contains details of all the data available through a fully searchable data dictionary and variable search tool http://www.bristol.ac.uk/alspac/researchers/our-data/.

When surveyed 8 weeks after the birth of their child, 13 351 women responded; 12 884 (96.5%) had partners, of whom more than 99% were identified as the father of the child.^22^ Mothers were given the option to involve their partner in the study and 8350 fathers responded. Our sample included 4492 mother–father–child trios with complete data: details are given below.

### Ethics statement

The authors assert that all procedures, including informed consent from all participants, contributing to this work comply with the ethical standards of the relevant national and institutional committees on human experimentation and with the Helsinki Declaration of 1975, as revised in 2008. All procedures involving human patients were approved by the ALSPAC Law and Ethics Committee and the Local Research Ethics Committees http://www.bristol.ac.uk/alspac/researchers/research-ethics/. Written informed consent was obtained from all participants.

### Measures

#### Depression symptoms

At child age 21 months, the Edinburgh Postnatal Depression Scale (EPDS) was completed by both parents.^23^ The EPDS is a ten-item assessment of symptoms in the past week, validated in mothers and fathers.^12,24^ Items include sadness: ‘I have felt sad or miserable’ and insomnia: ‘I have been so unhappy that I have had difficulty sleeping’. Items ‘I have looked forward with enjoyment to things’ and ‘I have been able to laugh and see the funny side of things’ were reverse coded. Item responses range from 1 to 4 (‘not at all’ to ‘most of the time’), a high total score indicates a more severe rating. Items, descriptive statistics and endorsement rates are reported in Table 1, Table 2, and Supplementary Table 1 available at https://doi.org/10.1192/bjp.2023.8.

**Table 1:** Depression items assessed for network analysis, their assigned label and community, with means, confidence intervals and reliability

Community and abbreviation	Item	Mean	95% CI	Cronbach's α
Father depression^a^				
d_anhedonia	I have looked forward with enjoyment to things	1.22	1.20–1.23	0.83
d_guilt	I have blamed myself unnecessarily when things went wrong	1.88	1.86–1.90	
d_worry	I have been anxious or worried for no good reason	1.65	1.62–1.67	
d_panic	I have felt scared or panicky for no very good reason	1.24	1.22–1.25	
d_overwhelm	Things have been getting on top of me	1.57	1.55–1.59	
d_insomnia	I have been so unhappy that I have had difficulty sleeping	1.17	1.16–1.18	
d_sadness	I have felt sad or miserable	1.43	1.41–1.45	
d_crying	I have been so unhappy that I have been crying	1.06	1.05–1.07	
d_harmIdeas	The thought of harming myself has occurred to me	1.06	1.05–1.07	
Mother depression^a^				
m_anhedonia	I have looked forward with enjoyment to things	1.27	1.25–1.28	0.86
m_guilt	I have blamed myself unnecessarily when things went wrong	2.08	2.05–2.10	
m_worry	I have been anxious or worried for no good reason	1.92	1.89–1.94	
m_panic	I have felt scared or panicky for no very good reason	1.46	1.43–1.48	
m_overwhelm	Things have been getting on top of me	1.86	1.84–1.88	
m_insomnia	I have been so unhappy that I have had difficulty sleeping	1.23	1.22–1.25	
m_sadness	I have felt sad or miserable	1.66	1.64–1.68	
m_crying	I have been so unhappy that I have been crying	1.39	1.37–1.41	
m_harmIdeas	The thought of harming myself has occurred to me	1.07	1.06–1.08	
Removed				
d_funny	I have been able to laugh and see the funny side of things	1.26	1.24–1.27	
m_funny	I have been able to laugh and see the funny side of things	1.33	1.31–1.34	

a. Depression items are taken from the Edinburgh Postnatal Depression Scale, assessed in parents at child age 21 months, range 1–4.

**Table 2:** Participant characteristics and comparisons between the analysis sample and the excluded sample

		Analysis sample (*N* = 4492)				Excluded sample (*N* = 10 049)				Difference test and effect size			
	Cronbach's^*a*^	Missing	Mean/*n* (%)	s.d.	Range	Missing	Mean/*n* (%)	s.d.	Range	*t*-test/*χ^2^*	*d.f.*	*P*	*d/h*
Social class	*−*	770	−	*−*	−	5745	−	*−*	−	244.9	5	<0.001	*−*
Professional	*−*	-	552 (14.8)	*−*	−	-	380 (8.8)	*−*	−	*−*	−	*−*	−
Managerial, technical	*−*	-	1780 (47.8)	*−*	−	-	1708 (39.7)	*−*	−	*−*	−	*−*	−
Skilled non-manual	*−*	-	999 (26.8)	*−*	−	-	1392 (32.3)	*−*	−	*−*	−	*−*	−
Skilled manual	*−*	-	310 (8.3)	*−*	−	-	718 (16.7)	*−*	−	*−*	−	*−*	−
Partly skilled	*−*	-	75 (2.0)	*−*	−	-	166 (3.9)	*−*	−	*−*	−	*−*	−
Unskilled	*−*	-	6 (0.0)	*−*	−	-	12 (0.0)	*−*	−	*−*	−	*−*	−
Father EPDS^a^	0.83	9	3.53	3.66	0–26	9336	4.01	4.22	0–27	4.08	2538	<0.001	0.13
Mother EPDS^a^	0.86	25	5.23	4.45	0–29	5295	6.09	5.02	0–30	9.14	9988.1	<0.001	0.18
Child emotional difficulties^b^	0.68	0	1.48	1.74	0–10	7483	1.58	1.81	0–10	2.36	7309.8	0.018	0.05
Child gender (female)	−	0	2204 (49.1)	−	−	306	5058 (48.9)	−	−	0.05	1	0.820	0.00

*d.f.* = degrees of freedom; *d/h* = Cohen's effect sizes.

a. Edinburgh Postnatal Depression Scale (EPDS) total score, assessed in parents at child age 21 months, range 0–30.

b. Child emotional symptoms subscale score from the Strengths and Difficulties questionnaire, assessed at age 9 years, range 0–10.

#### Child emotional difficulties

When the child was aged 9, 11 and 13 years the Strengths and Difficulties Questionnaire was completed by mothers.^25^ We used the five-item emotional difficulties subscale that assesses depression and anxiety symptoms, including ‘many worries, often seems worried’ and ‘often unhappy, down-hearted or tearful’. Subscale scores range from 0 to 10, high scores indicate more severe symptoms. Internal consistency was acceptable (α = 0.68 at each time point).

To examine the general burden of emotional symptoms in the transition to adolescence, while accounting for measurement error at different data-collection time points, we extracted a latent factor score of child emotional symptoms across time points. This allowed examination of the common variance of emotional symptoms across child ages.

Scores between time points correlated (*r*s = 0.49–0.59) and a Kaiser–Meyer–Olkin (KMO) test indicated that sampling adequacy was good (KMO = 0.70), the latent factor score was estimated (range: −1.02 to 5.24) using the lavaan R package.^26^

#### Demographics

Child gender was reported when the child was 8 weeks old. Social class was based on occupation and coded by ALSPAC according to the UK Office of National Statistics classification system of six categories: I, II, III non-manual, III manual, IV, V (I = professional and higher managerial, V = unskilled). Social class was reported by both parents at 18 weeks’ gestation, we created a household social class variable by selecting the highest report.

### The sample for analysis, demographics and missing data

We first constrained the sample to those with complete data for child emotional difficulties at age 9 years, which gave a potential sample of 7960 families. Of these, parent depression data were available for the final sample of 4492 families. Child emotional difficulties data were imputed using the mice R package.^27^ A full description of the missing data steps and plots are provided in Supplementary Fig. 1.

The analysis sample was compared with those excluded using *t*-tests, chi-square tests and Cohen's *d* and *h*-effect sizes, reported in Table 2. Child gender did not significantly differ between the groups. In the analysis sample compared with the excluded sample: there was a larger proportion of families in the highest two social class categories (I and II); children had lower levels of emotional difficulties at age 9 years (1.48 *v*. 1.58, *P* = 0.018) and mothers and fathers had lower depression scores (mothers 5.23 *v*. 6.09, fathers 3.53 *v*. 4.01, *P*s < 0.001). However, the effect sizes were very small (0.05, 0.13 and 0.18, respectively).

### Statistical analysis

All analyses were performed using R version 4.1.0.^28^

#### Symptom selection

If two items are highly correlated and both items have similar correlations with the rest of the symptoms within the network, they might represent the same underlying symptom, which may obscure other relationships within the network.^9^ Following the steps described in Levinson et al (2018),^14^ and reported in full in Supplementary Table 2, we identified overlapping dependent correlations in mother and father symptoms separately, using the goldbricker function in the networktools package.^29^ Four experienced researchers reviewed any identified pairs to ensure their pairing was theoretically meaningful.^14^

#### Network analysis and sample size

The network approach conceptualises mental disorders at the symptom level: symptoms are represented by nodes in the network and edges between nodes represent conditional associations, meaning the associations control for all other associations in the network (i.e. partial correlations). Networks were estimated using an unregularised Gaussian graphical model (GGM),^30^ which more reliably determines conditional associations with a high sample size and low-dimensional settings.^31^ We used the ggmModSelect function from the qgraph package,^32^ which selects the best GGM according to Bayesian information criterion. Owing to the ordinal, non-normally distributed data, we used Spearman's rank-correlations.

The Fruchterman–Reingold algorithm was used to plot symptoms with the strongest connections together at the centre of the graph.^33^ We did not include any thresholds for edge visualisation. As we were interested in the network structure, we examined network density (the number of estimated relative to the possible edges) and the average absolute edge weight.^34^

#### Symptom centrality, communities and bridge symptoms

The importance of each symptom within the overall network was assessed using the strength centrality index.^35^ We chose the strength index as it has been previously reported as conceptually meaningful, stable and replicable.^13,36,37^ Strength centrality describes how well a node is directly connected to other nodes, i.e. the absolute sum of the edge weights between one symptom and all other symptoms in the network. A full description of centrality indices is provided in Supplementary Table 3.

As we wanted to identify individual symptoms that ‘bridged’ the symptom networks between mothers and fathers, in network 1 we defined ‘communities of symptoms’ *a priori*: mother depression (ten mother EPDS items), father depression (ten father EPDS items).

Bridge symptoms assess the connections of each symptom to the community of symptoms outside its own (i.e. the influence of each mother symptom on the community of father symptoms and vice-versa). Bridge symptoms were assessed using the Bridge strength centrality index.^13^ Bridge expected influence, i.e. the symptom's cumulative influence outside of its own community, was used to identify the top 30% scoring symptoms, highlighted as ‘bridges’ in the network plot.^13^

We used the packages qgraph^32^ and networktools,^29^ for all estimates.

#### Network stability and replication

Stability is conceptually similar to the internal reliability of the network.^14^ Symptom centrality and bridge centrality indices were assessed for stability by estimating correlation stability (CS) coefficients, (estimates must be >0.25 to indicate that the centrality index is stable with values >0.5 preferred).^36^ Stability was also examined by estimating case-dropping subset bootstraps, which evaluate the maximum proportion of cases that can be dropped while the correlation between the original centrality indices and the new indices remains above 0.7.^36^ Edge weight stability was evaluated by bootstrapping 95% confidence intervals. We used the bootnet package for all stability estimates.^36^

Network replicability was assessed by halving the sample at random and comparing the network of each sample with 10 000 permutations. We used the network comparison test to examine total connectivity (i.e. the weighted sum of all the edges) using the global strength invariance test and the overall structure using the network structure invariance test.^38^

#### Analysis steps

We estimated two GGMs and the analysis proceeded in three steps. First, we used the initial model to examine the overall network structure of mother and father depression symptoms at child age 21 months. Second, we used the same model to identify bridges between mother and father symptoms. Third, we re-estimated the model including a child emotional difficulties factor score and examined associations with the parent symptom network. The analysis script is publicly available at https://doi.org/10.5281/zenodo.7409041.

## Results

### Symptom selection

Following the Goldbricker approach, we identified five overlapping pairs for mothers and one for fathers, reported in Supplementary Table 2. After reviewing these pairs, it was agreed that for four pairings the symptoms were conceptually independent (for example, insomnia and crying) and both were retained. The pairing of ‘unable to enjoy life’ and ‘unable to see the funny side of things’ was deemed to have conceptual overlap (representing anhedonia). We examined this pair in a network that included all the study variables (ten EPDS items for mothers and fathers and one child emotional difficulties score), where ‘unable to enjoy life’ was a bridge symptom and consequently was retained (the full network is presented in Supplementary Fig. 2). Therefore, we removed for both mothers and fathers: ‘unable to see the funny side of things’.

### Network stability and replication

For both networks, strength centrality indices were stable, the CS coefficient was 0.75 for network 1 and 0.52 for network 2, both above the stringent threshold for stability (CS > 0.50) and case-dropping bootstraps remained over 0.7.^36^ There were no negative edges in the network, therefore bridge expected influence is not reported because it is equivalent to bridge strength. Some edges were stable, but there was also considerable crossover between bootstrapped confidence intervals, therefore the rank order should be interpreted cautiously. Estimates and difference tests are reported in Supplementary Fig. 3–5.

Replicability tests demonstrated the validity of both networks: global strength and network structure did not differ significantly between the split half networks (network 1: strength_diff_ = 0.11, *P* = 0.430, edge_maxdiff_ = 0.09, *P* = 0.768; network 2: strength_diff_ = 0.13, *P* = 0.374, edge_maxdiff_ = 0.10, *P* = 0.586; distribution plots are reported in Supplementary Figure 6).

### Step 1: the network structure of parent symptoms

**Fig. 1:**
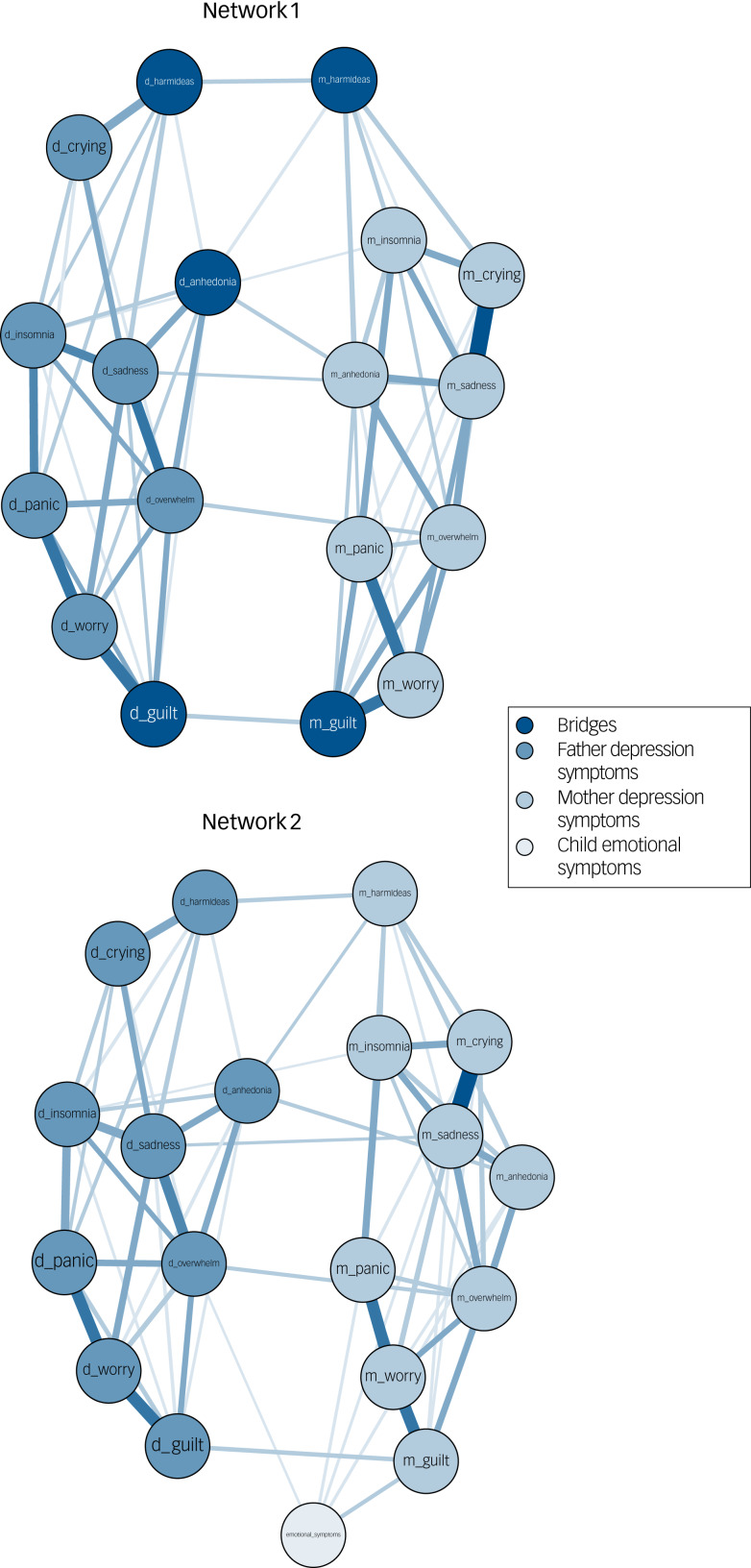
Network models: (a) network 1 and (b) network 2. Abbreviated terms are used in the figure; please see Table 1 for the full item details.

The first network is presented in Fig. 1(a), centrality indices and values are reported in Supplementary Fig. 7 and Supplementary Table 4. Partial correlation estimates are presented in full in Supplementary Fig. 8. The network density was 0.40 (61/153) with a mean edge weight of 0.05 (ranging from *r* = 0.04 (mother insomnia and father insomnia) to *r* = 0.41 (mother sadness and mother crying)). Mother and father symptoms showed high similarity in their patterns of associations within the network; the symptoms panic, worry and feeling guilty clustered in both mothers and fathers. Sadness and feeling overwhelmed were the most highly connected symptoms in the network for both mothers and fathers. Several symptoms had a significant edge with the same symptom in the other parent (specifically: insomnia, sadness, anhedonia, overwhelm, self-harm ideation and feelings of guilt (*r*s = 0.04–0.09)).

### Step 2: bridge symptoms between parent communities

We examined bridge symptoms in the first network, bridge centrality indices are reported in Supplementary Fig. 7. For both parents, feeling guilty and self-harm ideation were bridges, suggesting that these symptoms act as gateways between mother and father symptoms, each mutually reinforcing the other. For fathers, anhedonia was the most connected bridge symptom, providing a gateway to mother depression symptoms. Sensitivity analysis that included child gender and social class in the model did not change the magnitude or pattern of the associations and the bridge symptoms remained the same, the network is presented in Supplementary Fig. 9.

### Step 3: parent depression symptoms and child emotional difficulties

The second network is presented in Fig. 1(b), centrality indices and values are reported in Supplementary Fig. 10 and Supplementary Table 4. Partial correlation estimates are presented in full in Supplementary Fig. 8. The network density was 0.37 (66/171) with a mean weight of 0.05. The only bridge symptom connecting mother and father symptoms in the previous step that also associated with child emotional difficulties was feeling guilty in mothers (*r* = 0.09). The other mother symptoms that directly associated with child emotional difficulties were anhedonia, panic and sadness (*r*s = 0.04–0.06). In fathers, feeling overwhelmed was the only symptom that directly associated with child emotional difficulties. We also found evidence for indirect pathways to child emotional difficulties, where feeling guilty and anhedonia in fathers indirectly associated with child difficulties via the same symptoms in mothers. A sensitivity analysis was conducted that included child gender and social class in the model; the pattern and magnitudes of the associations were largely unchanged, presented in Supplementary Fig. 9.

## Discussion

This study used a network approach to examine the co-occurrence of depression symptoms between parents early in their child's development and associations with emotional difficulties in children at the transition to adolescence. We discuss the similarity of symptom clusters in parents, and highlight the importance of bridge symptoms as a reinforcing mechanism underlying the often-observed co-occurrence of depression in mothers and fathers. We then discuss parent-to-child symptom associations – which were not the same for each parent – and how the symptoms identified in this study may provide clinical targets for reducing transmission of depression within families.

### Main findings, comparison with other studies and interpretation

#### Overall network structure of mother and father symptoms

Our first aim was to examine the overall network structure of mother and father symptoms. By using network analysis, we found that symptoms intercorrelated with very similar clustering patterns in both parents. As expected, we found that panic, worry and feeling guilty clustered in mothers and fathers, supporting previous findings suggesting that these symptoms constitute an anxiety-related depression factor of the EPDS.^19^ Previous studies have found differences between mothers and fathers, positing a stronger role for anxiety-related depression symptoms for fathers,^39^ whereas, we found that the strength of the associations of the anxiety symptoms within the network were very similar for both parents. This may be because we measured symptoms when the infant was 21 months old. For fathers, anxiety symptoms have been found to increase prenatally, peak at birth and then rapidly reduce postnatally,^40^ suggesting that anxiety-related depression symptoms may be most salient for fathers early in the postnatal period.

As well as finding symmetry in symptom clusters between parents, we also found that the same symptoms in mothers and fathers had the greatest influence on the total network. For example, sadness and feeling overwhelmed were the most highly connected symptoms with the rest of the network for both parents. Of interest, we found that the same symptoms associated between mothers and fathers, suggesting that when specific symptoms are high in one parent, they are also high in the other parent, potentially contributing to the concordance of depression between parents.^4^

#### Symptoms bridging mother and father depression symptoms

Our second aim was to identify symptoms bridging mother and father depression symptoms, to provide insight into symptom-level mechanisms underlying the high rates of co-occurrence of parental depression.^4^ Our hypothesis was partially supported as we found that feeling guilty and self-harm ideation were bridge symptoms in both parents. This indicates that these symptoms act as a gateway, mutually activating and reinforcing the wider network of symptoms in the other parent.^13,41^ However, despite previous research finding that insomnia associated between mothers and fathers,^12^ we did not find that insomnia acted as a bridge symptom. The most likely explanation for this is because networks model conditional associations between groups of symptoms, highlighting the value of network modelling of complex relationships between individuals.

Importantly, these activating symptoms (feeling guilty and self-harm ideation) could be targeted for therapeutic deactivation. For example, one study examined whether reduction in the activation of influential symptoms would reduce overall activation of the grief symptom network. They found that reduced activation of influential symptoms more strongly associated with a greater reduction in overall network activation, compared with symptoms that were low in influence.^18^ The bridge symptoms identified in this study may therefore provide clinical targets when depression co-occurs between parents, by deactivating influential symptoms, thereby reducing co-activation of the wider network of symptoms between parents.

#### Role of bridge and other symptoms in the parent network associated with later emotional difficulties in the child

Our third aim was to examine whether the bridge and other symptoms within the parent network associated with later emotional difficulties in the child. In mothers, the bridge symptom feeling guilty directly associated with child emotional difficulties, as did panic, anhedonia and sadness. These results support previous findings that suggested anhedonia and impaired attention as potential mechanisms in the intergenerational transmission of depression.^16^ This may be explained in part by the impact of depression symptoms on mothers’ parenting,^17^ and the transmission of depressogenic cognitive styles from mothers to their children.^42^

For fathers, only the symptom feeling overwhelmed directly associated with child emotional symptoms. This is consistent with previous findings that indicators of being overwhelmed in fathers, such as ‘feeling trapped by my responsibilities as a parent’ was the strongest predictor of paternal depression severity.^43^ Indeed, our findings for aim one found feeling overwhelmed to be one of the most influential symptoms in the depression network for fathers. Therefore, it is plausible that this symptom is particularly important for overall depression severity in fathers, which in turn increases risk for emotional difficulties in children.

We also found evidence of mediated pathways from father symptoms to child emotional difficulties through the same symptom in mothers. This finding is reflected in existing literature where the effect of father depression is often mediated through other processes.^44^ One explanation may be that father depression can increase the negative impact of mother depression symptoms on children.^45^ Our finding that specific symptoms, when higher in one parent are also higher in the other parent, may provide new insight here. Of note, the symptom feeling guilty seems to play a particularly important role in familial transmission of depression, acting as a reinforcing bridge between parents, and providing a pathway from father to mother to child. These cascades of symptoms may present important targets for therapeutic deactivation, to reduce the transmission of depressive symptoms within families.

### Limitations

Some potential sources of bias should be noted. First, although it is plausible that parental depression in early life may lead to a home environment that has an impact on child symptoms, it is also likely that this pathway will reflect heritable influences. Assortative mating can result in genetic similarity between parents, potentially confounding parent associations.^46^ In addition, parents and their children are 50% genetically similar, therefore parent and child associations may be genetically confounded. Genetic confounding occurs because parent depression symptoms may be a marker of genetic predisposition, meaning that observed associations may reflect both environmentally and genetically mediated influences. Although previous estimates of the impact of genes on internalising symptoms have not been high,^47,48^ a genetically informative study investigating specific depression symptoms in families will be important to clarify and extend the findings presented here.

Second, emotional difficulties in children were rated by mothers. This can result in overestimated associations between parent and child depression symptoms when mothers are currently depressed,^49^ although it is not clear how this might have an impact on symptom-level associations.

Last, we examined a community sample. Associations between parent and child depression are particularly profound when parent symptoms are severe and persistent,^50^ therefore more complex patterns of associations may be found in clinical samples.

### Implications

In conclusion, by investigating mother and father depression at the symptom level, we identified bridge symptoms that may play a role in mutually reinforcing and activating the depression networks between parents. Child emotional difficulties directly associated with specific symptoms in mothers and indirectly with the same symptom in fathers. The symptom of feeling guilty both reinforced the mother and father symptom networks and provided a pathway from father to mother to child emotional difficulties. These symptoms may provide targets for therapeutic deactivation in interventions addressing the transmission of depression within families.

## Data Availability

ALSPAC data is available to researchers. Information regarding access can be found on the ALSPAC website (http://www.bristol.ac.uk/alspac/researchers/access/).

## Supplementary material

For supplementary material accompanying this paper visit https://doi.org/10.1192/bjp.2023.8. click here to view supplementary material
