# Supplementary material for: Mother and father depression symptoms and child emotional difficulties: a network model
Source: Br J Psychiatry. 2023 May;222(5):204–11. doi: 10.1192/bjp.2023.8 (PMC10895513; doi:10.1192/bjp.2023.8)
Supplement: Supplementary file 1 [file S0007125023000089sup001.docx]

**Mother and father depression symptoms and child emotional difficulties: a network model.**

Alex F. Martin, MSc^1^; Barbara Maughan, PhD^2^; Deniz Konac, MA^1,3^; Edward D. Barker, PhD^1^

^1^King’s College London, Department of Psychology, Institute of Psychiatry, Psychology & Neuroscience, London, UK; ^2^King’s College London, Social, Genetic & Developmental Psychiatry Centre, Institute of Psychiatry, Psychology and Neuroscience, London, UK; ^3^Department of Psychology, Adana Alparslan Turkes Science and Technology University, Adana, Turkey

**Supplementary**

**Table 1.** Item endorsement rates

**Table 2.** Depression symptom selection

**Table 3.** Description of centrality and bridge centrality indices

**Table 4.** Standardised and unstandardised strength centrality and bridge strength centrality values

**Supplementary**

**Figure 1.** Missing data plots and imputation

**Figure 2.** The network containing all study variables of interest

**Figure 3.** Edge stability and ranked order

**Figure 4.** Centrality and edge weight difference tests

**Figure 5.** Centrality stability using case-dropping bootstraps

**Figure 6.** Distributions of network invariance tests

**Figure 7.** Centrality and bridge centrality plots for network 1

**Figure 8**. Networks 1 and 2 including partial correlation statistics

**Figure 9.** Sensitivity analysis including covariates in the models

**Figure10.** Centrality plot for network 2

**Tables**

| **Table1.** Item endorsement rates | | | | | | | | | | | |
| --- | --- | --- | --- | --- | --- | --- | --- | --- | --- | --- | --- |
|  | **1** | | **2** | | **3** | | **4** | | **Missing** | | **Final N** |
|  | *N* | % | *N* | % | *N* | % | *N* | % | *N* | % |  |
| d_funny | 3519 | 78.4% | 812 | 18.1% | 140 | 3.1% | 20 | 0.4% | 1 | 0.0% | 4491 |
| d_anhedonia | 3661 | 81.5% | 694 | 15.5% | 113 | 2.5% | 22 | 0.5% | 2 | 0.0% | 4490 |
| d_crying | 4243 | 94.5% | 228 | 5.1% | 16 | 0.4% | 3 | 0.1% | 2 | 0.0% | 4490 |
| d_guilt | 1614 | 35.9% | 1921 | 42.8% | 838 | 18.7% | 119 | 2.6% | 0 | 0.0% | 4492 |
| d_harmIdeas | 4292 | 95.5% | 142 | 3.2% | 52 | 1.2% | 6 | 0.1% | 0 | 0.0% | 4492 |
| d_insomnia | 3867 | 86.1% | 488 | 10.9% | 124 | 2.8% | 11 | 0.2% | 2 | 0.0% | 4490 |
| d_overwhelm | 2433 | 54.2% | 1582 | 35.2% | 447 | 10.0% | 30 | 0.7% | 0 | 0.0% | 4492 |
| d_panic | 3666 | 81.6% | 614 | 13.7% | 183 | 4.1% | 29 | 0.6% | 0 | 0.0% | 4492 |
| d_sadness | 2843 | 63.3% | 1400 | 31.2% | 223 | 5.0% | 24 | 0.5% | 2 | 0.0% | 4490 |
| d_worry | 2559 | 57.0% | 1045 | 23.3% | 813 | 18.1% | 75 | 1.7% | 0 | 0.0% | 4492 |
| m_funny | 3275 | 73.0% | 980 | 21.9% | 214 | 4.8% | 16 | 0.4% | 7 | 0.2% | 4485 |
| m_anhedonia | 3462 | 77.1% | 870 | 19.4% | 135 | 3.0% | 21 | 0.5% | 4 | 0.1% | 4488 |
| m_crying | 2952 | 65.8% | 1350 | 30.1% | 169 | 3.8% | 18 | 0.4% | 3 | 0.1% | 4489 |
| m_guilt | 1242 | 27.7% | 1865 | 41.5% | 1181 | 26.3% | 203 | 4.5% | 1 | 0.0% | 4491 |
| m_harmIdeas | 4282 | 95.3% | 134 | 3.0% | 64 | 1.4% | 11 | 0.2% | 1 | 0.0% | 4491 |
| m_insomnia | 3676 | 81.9% | 599 | 13.3% | 198 | 4.4% | 18 | 0.4% | 1 | 0.0% | 4491 |
| m_overwhelm | 1573 | 35.0% | 2026 | 45.1% | 848 | 18.9% | 45 | 1.0% | 0 | 0.0% | 4492 |
| m_panic | 3088 | 68.8% | 860 | 19.2% | 439 | 9.8% | 103 | 2.3% | 2 | 0.0% | 4490 |
| m_sadness | 2100 | 46.8% | 1881 | 41.9% | 460 | 10.2% | 50 | 1.1% | 1 | 0.0% | 4491 |
| m_worry | 1928 | 43.0% | 1152 | 25.7% | 1259 | 28.1% | 148 | 3.3% | 5 | 0.1% | 4487 |

*Note.* Sample N = 4492; items are from the Edinburgh Postnatal Depression Scale (EPDS), assessed at child age 21 months, items range 1-4,

d_ indicates father rated symptom, m_ indicates mother rated symptom

**Table 2.** Depression symptom selection

|  | **Item pairs** | **Proportion** |
| --- | --- | --- |
| Father depression | anhedonia, **funny** | 0.17 |
| Mother depression | panic, guilt | 0.11 |
|  | anhedonia, **funny** | 0.17 |
|  | worry, panic | 0.17 |
|  | worry, guilt | 0.22 |
|  | crying, insomnia | 0.28 |

*Note.* Proportion matrix for item pairs with less than 30% of significantly different correlations, significance = p < .001. Items removed are bolded.

**Symptom selection**

If two items are highly correlated, this suggests that they might represent the same underlying symptom, and this may obscure other relationships within the network (Fried et al. 2017). To test this, we examined mother and father symptoms separately using dependent correlation analysis, where the correlation between two items is examined alongside the correlations between each item in the pair and the rest of the nodes within the network. If two highly correlated items measure the same construct, they will have similar correlations with the rest of the nodes within the network, and one can be dropped. Following the steps described in Levinson et al. (2018), we combined a theoretical and data-driven approach: we used the Goldbricker approach to identify overlapping dependent correlations and four experienced researchers reviewed any identified pairs to ensure their pairing was theoretically meaningful. Additionally, we examined a network which included all the study variables (21 nodes: ten Edinburgh Postnatal Depression Scale (EPDS) items for mothers and fathers and one child outcome) to ensure that we did not remove any symptoms which played a central role within the network. A large sample size increases the chance of variables appearing unique, therefore we decided a priori to use stringent cut offs for the proportion of correlations which were different between the pair (0.40) and the significance threshold (p < .001). We used the goldbricker function in the R package networktools.(33)

**References**

Fried EI, Cramer AOJ. Moving Forward: Challenges and Directions for Psychopathological Network Theory and Methodology. Perspectives on Psychological Science. 2017; 12(6): 999-1020

Levinson CA, Brosof LC, Vanzhula I, Christian C, Jones P, Rodebaugh TL, et al. Social anxiety and eating disorder comorbidity and underlying vulnerabilities: Using network analysis to conceptualize comorbidity. Int J Eat Disord. 2018; 51(7): 693-709.

Jones P. networktools: Tools for Identifying Important Nodes in Networks. R package. 2020

**Table 3.** Description of centrality and bridge centrality indices

| Index | Brief description | Detail |
| --- | --- | --- |
| Strength | Quantifies how well a node is directly connected to other nodes | The absolute sum of the edge weights between one node and all other nodes in the network |
| Bridge strength | Quantifies how well a node is directly connected to other nodes outside of its own community | The absolute sum of the edge weights between one node and all other nodes outside of the node's own community |
| Bridge expected influence | Quantifies how well a node is directly connected to other nodes outside of its own community, accounting for positive and negative edges (i.e. positive connectivity) | The cumulative sum of the edge weights between one symptom and all other symptoms in the network outside of the node's own community |

*Note.* The network approach conceptualises mental disorders at the symptom level: symptoms are represented by nodes in the network and edges between nodes represent conditional associations. Communities of nodes are defined a priori, i.e. are not based on the outcome of network analysis.

**Centrality indices** are taken from Epskamp, S., Borsboom, D., & Fried, E. I. (2018). Estimating psychological networks and their accuracy: A tutorial paper. *Behavior Research Methods*, *50*(1), 195-212. <https://doi.org/10.3758/s13428-017-0862-1> and McNally, R. J. (2016). Can network analysis transform psychopathology? *Behaviour Research and Therapy*, *86*, 95-104. <https://doi.org/https://doi.org/10.1016/j.brat.2016.06.006>

**Bridge centrality indices** are taken from Jones, P. J., Ma, R., & McNally, R. J. (2019). Bridge Centrality: A Network Approach to Understanding Comorbidity. *Multivariate Behav Res*, 1-15. <https://doi.org/10.1080/00273171.2019.1614898>

| **Table 4.** Standardised and unstandardised strength centrality and bridge strength centrality values | | | | |  |
| --- | --- | --- | --- | --- | --- |
| Item | **Network 1 centrality** | | **Network 2 centrality** | |  |
|  | Standardised strength values | Unstandardised strength values | Standardised strength values | Unstandardised strength values |  |
| d_anhedonia | -0.83 | 0.68 | -0.59 | 0.68 |  |
| d_crying | -1.61 | 0.54 | -1.22 | 0.54 |  |
| d_guilt | -0.32 | 0.77 | -0.18 | 0.77 |  |
| d_harmIdeas | -1.37 | 0.58 | -1.03 | 0.58 |  |
| d_insomnia | -0.02 | 0.82 | 0.06 | 0.82 |  |
| d_overwhelm | 1.07 | 1.02 | 1.11 | 1.05 |  |
| d_panic | 0.14 | 0.85 | 0.20 | 0.85 |  |
| d_sadness | 1.68 | 1.13 | 1.44 | 1.13 |  |
| d_worry | 0.43 | 0.91 | 0.43 | 0.91 |  |
| m_anhedonia | -0.53 | 0.73 | -0.15 | 0.78 |  |
| m_crying | 0.38 | 0.90 | 0.39 | 0.90 |  |
| m_guilt | 0.10 | 0.85 | 0.47 | 0.91 |  |
| m_harmIdeas | -1.61 | 0.54 | -1.23 | 0.54 |  |
| m_insomnia | -0.32 | 0.77 | -0.18 | 0.77 |  |
| m_overwhelm | 0.70 | 0.95 | 0.63 | 0.95 |  |
| m_panic | -0.15 | 0.80 | 0.18 | 0.85 |  |
| m_sadness | 1.96 | 1.18 | 1.80 | 1.21 |  |
| m_worry | 0.27 | 0.88 | 0.29 | 0.87 |  |
| emotional_symptoms | na | na | -2.42 | 0.28 |  |

**Figures**


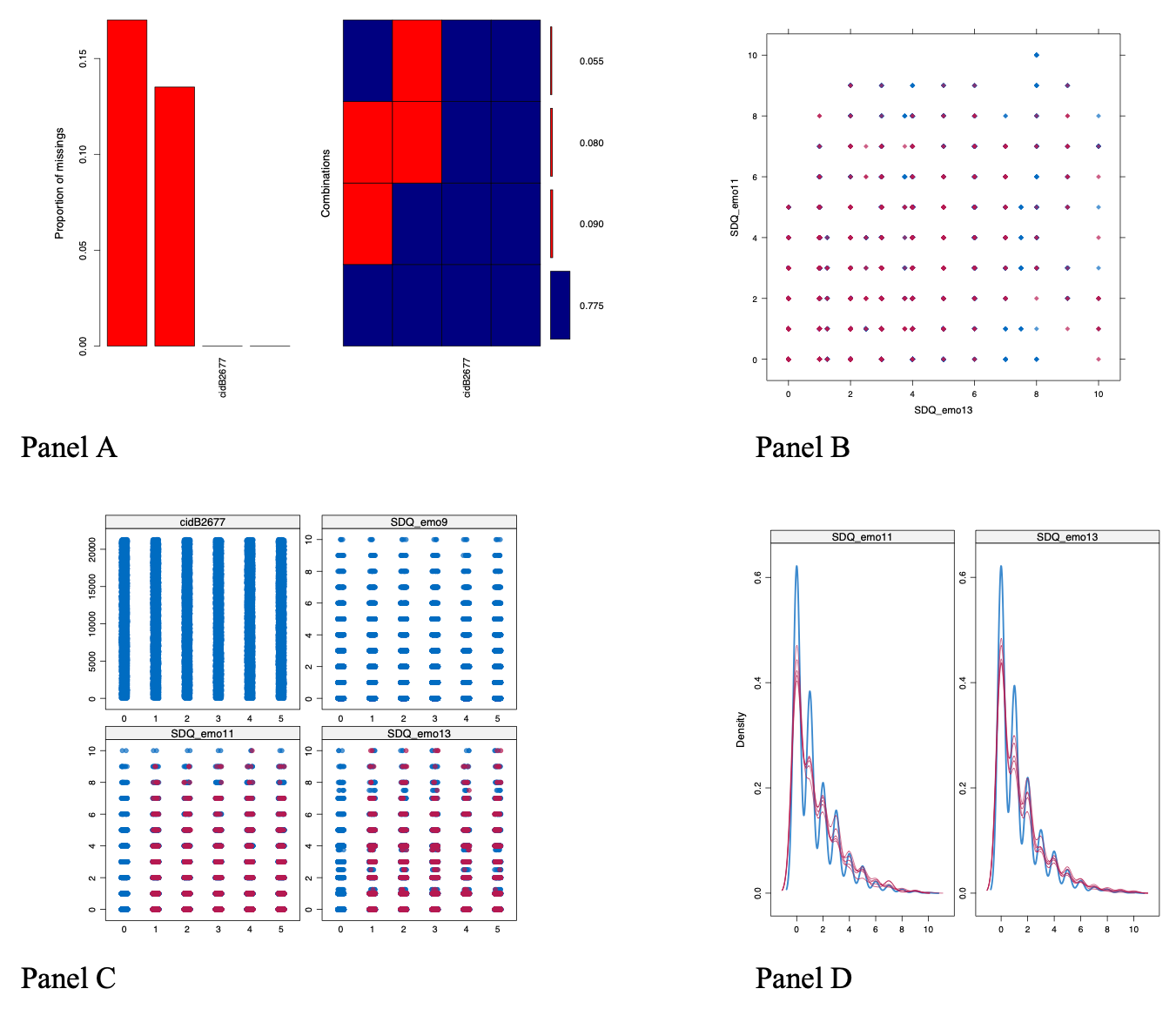


**Figure 1.** Missing data plots and imputation

Panel A: Missing data patterns for SDQ emotional difficulty total scores at age 11 and 13, missing = red

Panel B: Scatterplot comparing the distribution of SDQ emotional difficulty total scores at ages 11 and 13, in the observed (blue points) and imputed (red points) data

Panel C: Stripplot comparing the distribution of scores as individual points for SDQ emotional difficulty total scores at ages 9, 11, and 13, in the observed (blue points) and imputed (red points) data

Panel D: Densityplot comparing the density of SDQ emotional difficulty total scores at ages 11 and 13, in the observed (blue points) and imputed (red points) data

*Note.* cidB2677 = participant ID, excluded as a predictor in the imputation matrix

In each plot, in panels B-D the shape of the imputed points (red) is very similar to the observed points (blue), suggesting that the imputed values are plausible values.

**Process**

We first constrained the sample to those with complete data for child emotional difficulties at age 9, which gave a potential sample of 7,960 families

Of these, parent depression data were missing (i.e. missing more than two items) for 52 (0.7%) mothers, 2,522 (31.7%) fathers, and 894 (11.2%) mother and father pairs. With a large total proportion of missing parent depression data (43.6%) and disproportionately high missing father data, imputation of depression data was not deemed appropriate (Little & Rubin, 2019). We therefore excluded any families where mothers and/or fathers were missing more than two depression items, giving a final sample of 4,492 families.

Emotional difficulties data were missing for 607 (14%) children at age 11 and 764 (17%) at age 13. Missing data were imputed using the mice R package, using five imputed data sets and the predictive mean matching method, which restricts imputations to the observed values (van Buuren et al., 2011). The distributions and density of the observed and imputed data were highly similar (as seen in the plots above), suggesting that the imputation values were plausible values.

**References:**

Epskamp S, Fried EI. A tutorial on regularized partial correlation networks. Psychological Methods. 2018; 23(4): 617-34.

Little RJA, Rubin DB. Statistical analysis with missing data. John Wiley & Sons, 2019.

van Buuren, S., & Groothuis-Oudshoorn, K. (2011). mice: Multivariate Imputation by Chained Equations in R. *Journal of statistical software*, *45*(3). <https://doi.org/10.18637/jss.v045.i03>

For a helpful example with R code: Michy, A. (2018). *Imputing Missing Data with R; MICE package*. datascience+. Retrieved Sept 2021 from <https://datascienceplus.com/imputing-missing-data-with-r-mice-package/>

**Figure 2.** The network containing all study variables of interest

*Note.* 10 father depression items and 10 mother depression items at child age 21 months specified as separate communities, 1 child emotional difficulties factor score across ages 9, 11, and 13, specified as a community; Communities: blue = mother, green = father; Bridges = orange, bridge proportion threshold = 0.7; Blue lines = positive correlations, thicker lines = stronger correlations.

**Figure 3.** Edge stability and ranked order

*Note.* Based on 95% bootstrapped confidence intervals of the edge weights. Network 1 (left) and 2 (right)

**Figure 4.** Centrality and edge weight difference tests

*Note.* Based on 1000 bootstraps. Network 1 (upper) and 2 (lower). Grey squares indicate non-significant differences, black squares indicate significant differences. Left to right: edge-, strength-, bridge strength- difference plots.

**Figure 5.** Centrality stability using case-dropping bootstraps

*Note.* Network 1 (left) and 2 (right). Points = mean of 1000 bootstrapped correlations for centrality value between sample and subsample after dropping x proportion of cases; shaded area = 95% confidence intervals; bridge centrality indices were only estimated for network 1.

**Figure 6.** Distributions of network invariance tests

*Note.* Based on 10,000 permutations. Network 1 (upper) and network 2 (lower). Difference in global strength (left panel); maximum difference in edge strength (right panel).

**Figure 7.** Centrality and bridge centrality plots for network 1

*Note.* The x-axis indicates raw scores; greater scores = more central, ordered from strongest to weakest item.

**Figure 8.** Networks 1 (left) and 2 (right) including partial correlation statistics

**Figure 9.** Sensitivity analysis including covariates in the models

*Note.* Network 1 (left) and 2 (right). Covariates = child sex and social class

**Figure 10.** Centrality plot for network 2

*Note*. The x-axis indicates raw scores; greater scores = more central, ordered from strongest to weakest item.
